# Advancing States Through the OAA Reauthorization

**DOI:** 10.1093/geroni/igaa057.2573

**Published:** 2020-12-16

**Authors:** Damon Terzaghi

**Affiliations:** ADvancing States, Arlington, Virginia, United States

## Abstract

States are major policymakers and funders of community-based services for older Americans and ADvancing States played a significant role in supporting the OAA authorization this year. This presentation will provide the states' perspective regarding funding, services, research and demonstrations, and more.

